# Community pharmacist-administered seasonal influenza vaccination: a national customer survey

**DOI:** 10.1186/s40545-020-00259-7

**Published:** 2020-09-25

**Authors:** Dominik Stämpfli, Adrian Martinez-De la Torre, Sophie Du Pasquier, Danielle Stegmann, Andrea Brügger, Andrea M. Burden

**Affiliations:** 1 Pharmacoepidemiology, Institute of Pharmaceutical Sciences, Department of Chemistry and Applied Biosciences, Swiss Federal Institute of Technology, ETH Zurich, Vladimir-Prelog-Weg 4, CH-8093 Zurich, Switzerland; 2 Community Pharmacy, Center for Primary Care and Public Health (Unisanté), University of Lausanne, Lausanne, Switzerland; 3 Swiss Association of Pharmacists, Liebefeld, Switzerland

**Keywords:** Seasonal influenza, Vaccination, Satisfaction, Survey, Community pharmacy

## Abstract

**Background:**

In Switzerland, the influenza vaccination is recommended for high-risk groups and people who have contact with high-risk groups. Since 2015, Swiss pharmacists are allowed to vaccinate healthy adults after acquiring a certificate of competence for vaccination and blood sampling techniques. We aimed to assess customers of the seasonal influenza vaccination in pharmacies in regard to their satisfaction, motivation, and reasons.

**Methods:**

Swiss pharmacies collected survey data during a period of 12 weeks from mid-October 2019 to mid-January 2020. Each participating pharmacy was sent 20 questionnaires to be handed out to vaccinated customers. The questionnaire was available in German and French and subdivided into four sections: demographic information, satisfaction, reasons for getting the vaccination, and reasons for choosing a pharmacy as a place of vaccination. We tested for statistical differences in answer tendencies across strata on questionnaire language, age groups, and levels of education.

**Results:**

Of the 1600 surveys sent, 80 pharmacies sent back 656 completed questionnaires (return rate, 41%). Main age bracket was 65–74 years (26.2%), followed by 55–64 years (24.7%), with an equal distribution of reported sex (female, 49.5%). Of the respondents, 99% would have recommended the service and 88.5% felt very comfortable being vaccinated by a pharmacist. Satisfaction included injection technique, used facilities, preparatory discussions, and pricing of the service. Easy scheduling was a main motivation for choosing a pharmacy as the vaccination provider. We identified minor differences in answer tendencies across questionnaire language and age groups, but not across levels of education.

**Conclusion:**

Customer satisfaction with community pharmacist-administered seasonal influenza vaccinations is high in Switzerland.

## Main body

### Introduction

Globally, influenza is a major cause of morbidity and mortality, with 291,243 to 645,832 estimated seasonal influenza-associated respiratory deaths annually [1]. With vaccination being the most effective preventive measure against influenza [2], the Federal Office of Public Health of Switzerland (FOPH) is recommending the vaccination for people with an increased risk of complications of influenza and for people who have contact with these persons within the family or during their private or professional activities [3]. In this context, people with an increased risk of complications include persons over 65 years of age, pregnant women, women who have given birth in the last 4 weeks, premature babies from the age of 6 months for the first two winters after birth, persons with certain chronic diseases (from the age of 6 months), and patients in nursing homes and in facilities for patients with chronic diseases [3]. In Switzerland, the FOPH estimated that 209,200 people (around 2.5% of the Swiss population) consulted a general practitioner for a flu-like illness in 2019 [4]. Thus, the influenza vaccination may, additionally, be considered for persons who wish to reduce their own risk of influenza for private or professional reasons [3].

Increasing the influenza vaccination rate in the population may result in a reduction of influenza cases and a subsequent reduction in healthcare utilisation and costs. In recent decades, a number of countries have expanded the role of pharmacists to include providing influenza vaccinations [5]. As of 2015, Swiss pharmacists are allowed to vaccinate healthy adults after successfully completing a formal training and acquiring a certificate of competence for vaccination and blood sampling techniques [6]. Since May 2015, 2060 Swiss pharmacists have acquired this certificate of competence [7]. Additionally, pharmacies need to apply for vaccination permits at cantonal level [8]. At present, the seasonal influenza vaccination and selected cantonally defined other vaccinations are possible in a total of 22 of 26 cantons, whereas only the canton of Tessin requires a physician’s prescription [9]. While this service is not covered by the Swiss compulsory basic health insurance, this initiative aims to increase accessibility to influenza vaccines. In the 2017/2018 influenza season, 15,617 of 1,152,000 delivered vaccine doses (1.4%) were delivered in pharmacies [10]. It is estimated that these community pharmacy-administered vaccinations accounted for net cost savings of CHF 143,021 for the Swiss economy [10].

To date, evidence on customer satisfaction and motivation for a pharmacist-administered seasonal influenza vaccination is scarce [11–13]. Thus, the aim of this study was to identify customer demographics and gain insights into the motivation and satisfaction with Swiss community pharmacist-administered seasonal influenza vaccinations.

### Methods

#### Study design

Survey data was collected by voluntarily participating Swiss pharmacies during a period of 12 weeks from mid-October 2019 to mid-January 2020. Based on a participation rate of a previous Swiss study [14], we randomly selected 526 Swiss pharmacies to be asked to participate in the survey. Randomisation was performed on cantonal level to obtain a representative sample of pharmacies across Switzerland. Pharmacies were asked to sign up for participation through an online form generated by the Swiss Association of Pharmacists (pharmaSuisse). Each participating pharmacy was sent a bundle of 20 questionnaires and 20 sealable envelopes alongside written instructions and a pre-stamped return envelope. Pharmacists were asked to hand vaccinated customers one questionnaire and one sealable envelope. Participation in the survey by the customers was voluntary, and consent was considered to be given when the questionnaire was filled. Customers were asked to place their completed questionnaires in the sealable envelopes to hide their answers from the pharmacies. To ensure the anonymity of, both, customers and pharmacies, the only identifier included in the questionnaire was the four-digit zip codes indicating the commune of the vaccinating pharmacy. Data collection was terminated by recalling questionnaire bundles at the end of the data collection period.

#### Questionnaire

The electronically evaluable questionnaire was designed in German and translated into French by two independent translators. The German version of the questionnaire is displayed in Additional Figure 1. The questionnaire was subdivided into four sections: demographic information (i.e., zip code of pharmacy, sex [as salutation], age category, highest level of education, number of past vaccinations), satisfaction (technique, discussion, premises, pricing, recommendation), reasons for getting the vaccination (protection of oneself or others, professional risk for oneself or other), and reasons for choosing a pharmacy (opening hours, past experiences, availability of primary care physician, pricing, trust, accessibility, appointment). Wherever appropriate, a Likert scale with six plus one fully labelled levels was used: the six labels ranged from “does not apply at all” to “does fully apply;” we additionally included an option “not answerable” on the right side [15]. Questions were phrased to allow for a consistent use of the same labels throughout the questionnaire. The questionnaire was electronically enabled and evaluated by using TeleForm [16].

#### Statistical analysis

Survey data was aggregated and analysed using descriptive statistics. Zip codes were amended information on spatial divisions and typologies with data of the Swiss Federal Statistical Office [17]. Demographic characteristics were summarised using means and standard deviations and counts and proportions where appropriate, stratified by questionnaire language (German vs. French). Differences in demographic characteristics between the questionnaire languages were assessed using *t* test and chi-square test for continuous and categorical variables, respectively. Likert scales were summarised using counts and proportions. “Not answerable” responses and missing data were omitted from the analysis.

In secondary analyses, we stratified the Likert scales by levels of education, questionnaire language, and two age groups of respondents (< 65 years, ≥ 65 years). We used a Kruskal-Wallis test to check for differences in responses across levels of education. Mann-Whitney *U* test was used to compare responses between questionnaire language and the two age groups. The significance level was set at 5%. All analyses were performed in R [18] with the additional packages gglot2 [19], dplyr [20], data.table [21], and RSwissMaps [22].

### Results

Of the 526 pharmacies which were asked to participate in the survey, 80 pharmacies (15.2%) signed up for data collection. A total of 1600 questionnaires were sent in bundles of twenty to 52 German-speaking and 28 French-speaking pharmacies. At the end of the 12 weeks of survey data collection, 520 completed German questionnaires and 136 completed French questionnaires were returned, equalling in an overall return rate of 41% (50% German-speaking, 24% French-speaking). Two French questionnaires received from the Italian language region were included as coming from the French language region.

**Fig. 1:**
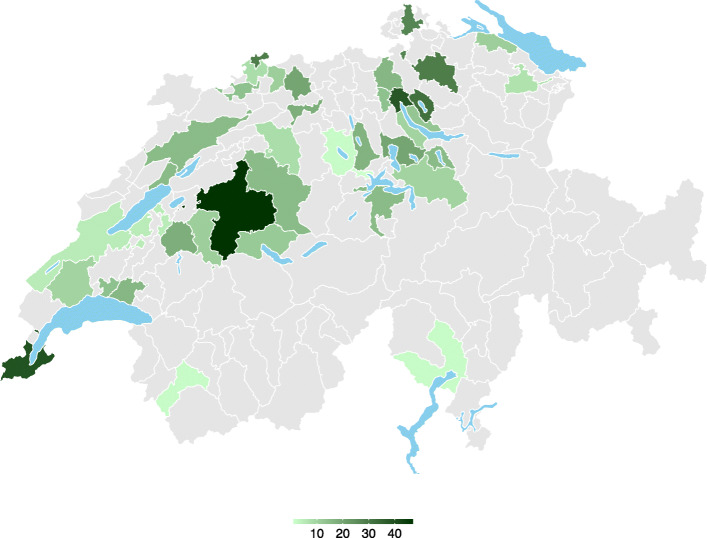
Questionnaires answered by Swiss districts. Colour density by numbers of questionnaires

Figure 1 displays survey data origin based on Swiss districts. Data included *n* = 636 (97%) questionnaires from pharmacies in urban communes and *n* = 20 (3%) questionnaires from sub-urban communes.

Table 1 shows the demographic data of the customer population. Main age bracket was 65–74, followed by 55–64. Female sex was reported in 49.5% of questionnaires. Reported highest acquired level of education was mainly higher education (i.e., universities, universities of applied sciences; 38.9%).

**Table 1:** Characteristics of survey respondents

	Overall		German*		French*		*p* value^†^
	(*n* = 656)		(*n* = 521)		(*n* = 135)		
	*N*	(%)	*N*	(%)	*N*	(%)	
Female	309	(47.1)	245	(49.4)	64	(47.4)	0.594
Missing					9	(6.7)	
Age							0.57
< 16	1	(0.2)	1	(0.2)	0	(0.0)	
16–17	0	(0.0)	0	(0.0)	0	(0.0)	
18–24	9	(1.4)	6	(1.2)	3	(2.2)	
25–34	31	(4.7)	25	(4.8)	6	(4.4)	
35–44	100	(15.2)	74	(14.2)	26	(19.3)	
45–54	101	(15.4)	85	(16.3)	16	(11.9)	
55–64	160	(24.4)	127	(24.4)	33	(24.4)	
65–74	170	(25.9)	135	(25.9)	35	(25.9)	
75–84	70	(10.7)	60	(11.5)	10	(7.4)	
> 85	6	(0.9)	4	(0.8)	2	(1.5)	
Missing	8	(1.2)	4	(0.8)	4	(3.0)	
Education							0.007
Compulsory school	34	(5.2)	29	(5.6)	5	(3.7)	
Secondary: vocational	191	(29.1)	151	(29.0)	40	(29.6)	
Secondary: general school	43	(6.6)	34	(6.5)	9	(6.7)	
Higher vocational	123	(18.8)	110	(21.1)	13	(9.6)	
Higher education	249	(38.0)	188	(36.1)	61	(45.2)	
Missing	16	(2.4)	9	(1.7)	7	(5.2)	
Vaccination elsewhere							0.116
No	36	(5.5)	26	(5.0)	10	(7.4)	
I do not know	56	(8.5)	39	(7.5)	17	(12.6)	
Yes	550	(83.8)	446	(85.6)	104	(77.0)	
Missing	14	(2.1)	10	(1.9)	4	(3.0)	
Previous pharmacy influenza vaccination							0.801
0	268	(40.9)	208	(39.9)	60	(44.4)	
1–2	198	(30.2)	157	(30.1)	41	(30.4)	
3–4	108	(16.5)	90	(17.3)	18	(13.3)	
5	68	(10.4)	55	(10.6)	13	(9.6)	
Missing	14	(2.1)	11	(2.1)	3	(2.2)	
Previous physician influenza vaccination							0.337
0	213	(32.5)	171	(32.8)	42	(31.1)	
1–2	144	(22.0)	110	(21.1)	34	(25.2)	
3–4	84	(12.8)	70	(13.4)	14	(10.4)	
5	197	(30.0)	153	(29.4)	44	(32.6)	
Missing	18	(2.7)	17	(3.3)	1	(0.7)	
Another vaccine at pharmacy (yes)	86	(13.1)	68	(13.1)	18	(13.3)	0.993
Missing	14	(2.1)	11	(2.1)	3	(2.2)	

*Refers to questionnaire language

^†^Numerical and binary variables were compared using *t* test and categorical variables with chi-square test

**Fig. 2:**
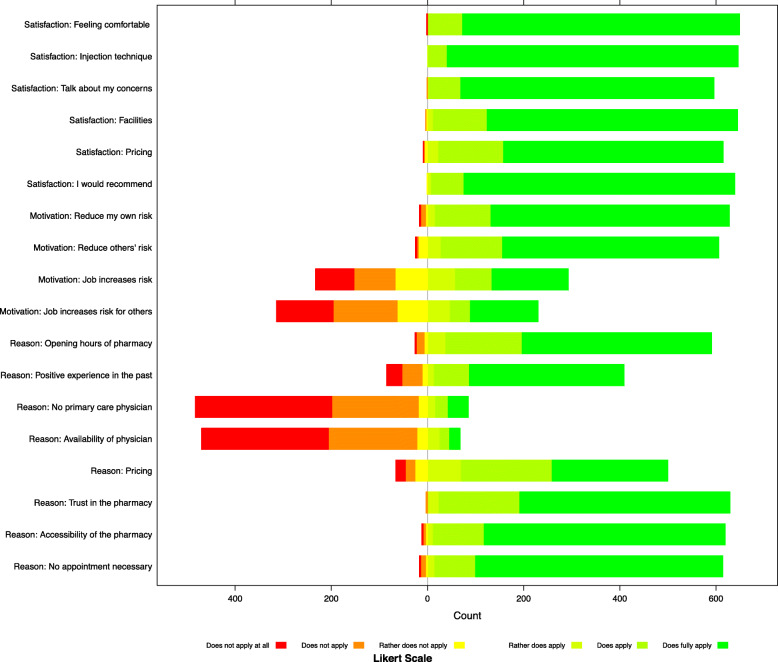
Likert scale answers for all main questions

Results of the main Likert scale questionnaire items are visualised in Fig. 2 and reported in Table 2. Over 95.0% (*n* = 615) of the respondents were satisfied with the pricing of the vaccination and 98.9% (639) would recommend getting the seasonal influenza vaccination in the pharmacy. The main motivational factors to receive an influenza vaccination were to reduce one’s own risk (*n* = 628, 97.0%) and to reduce others’ risk (607, 95.4%). Comparatively, risks associated with the occupation were less frequently mentioned as a decisive factor but accounted for 47.0% (*n* = 293, ‘job increases own risk’) and 37.0% (230, ‘job increases risks for others’) of responses. For 76.6% of the respondents, the fact of not having a primary care physician, and for 75.6% of the clients, the availability of a physician to conduct the vaccination was not seen as a reason for vaccinating in the pharmacy. No need for an appointment was seen as a relevant factor as 96.2% of the respondents answered positively. Additionally, 92.6 and 98.3% of respondents stated opening hours of the pharmacies and trust to the pharmacy as factors for choosing a pharmacy as a place to get vaccinated. A positive previous experience was stated by 410 (65.0%) respondents as a reason for vaccinating in the pharmacy, while only 374 (57.0%) respondents had answered having had done a previous influenza vaccination in a pharmacy.

**Table 2:** Likert scale counts and proportions for all main questions

	Does not apply at all		Does not apply		Rather does not apply		Rather does apply		Does apply		Does fully apply		Not Answerable	
Satisfaction: Feeling comfortable	2	0.31%	0	0.00%	0	0.00%	2	0.31%	71	10.91%	576	88.48%	0	0.00%
Satisfaction: Injection technique	0	0.00%	0	0.00%	0	0.00%	2	0.31%	39	6.02%	605	93.36%	2	0.31%
Satisfaction: Talk about my concerns	0	0.00%	1	0.15%	0	0.00%	3	0.46%	66	10.22%	527	81.58%	49	7.59%
Satisfaction: Facilities	0	0.00%	1	0.15%	3	0.46%	12	1.85%	112	17.23%	521	80.15%	1	0.15%
Satisfaction: Pricing	1	0.15%	2	0.31%	6	0.93%	23	3.55%	135	20.87%	457	70.63%	23	3.55%
Satisfaction: I would recommend	0	0.00%	0	0.00%	1	0.16%	8	1.24%	68	10.54%	563	87.29%	5	0.78%
Motivation: Reduce my own risk	3	0.46%	10	1.55%	4	0.62%	16	2.47%	116	17.93%	496	76.66%	2	0.31%
Motivation: Reduce others’ risk	3	0.47%	3	0.47%	19	2.99%	28	4.40%	128	20.13%	451	70.91%	4	0.63%
Motivation: Job increases risk	80	12.84%	86	13.80%	67	10.75%	58	9.31%	76	12.20%	159	25.52%	97	15.57%
Motivation: Job increases risk for others	118	19.00%	133	21.42%	63	10.14%	47	7.57%	42	6.76%	141	22.71%	77	12.40%
Reason: Opening hours of pharmacy	3	0.47%	16	2.51%	7	1.10%	38	5.96%	159	24.92%	394	61.76%	21	3.29%
Reason: Positive experience in the past	32	5.07%	42	6.66%	11	1.74%	14	2.22%	74	11.73%	322	51.03%	136	21.55%
Reason: No primary care physician	284	45.01%	180	28.53%	19	3.01%	17	2.69%	26	4.12%	42	6.66%	63	9.98%
Reason: Availability of physician	264	42.51%	184	29.63%	22	3.54%	26	4.19%	20	3.22%	22	3.54%	83	13.37%
Reason: Pricing	20	3.17%	20	3.17%	26	4.12%	70	11.09%	189	29.95%	241	38.19%	65	10.30%
Reason: Trust in the pharmacy	0	0.00%	3	0.47%	0	0.00%	24	3.75%	168	26.25%	437	68.28%	8	1.25%
Reason: Accessibility of the pharmacy	3	0.47%	5	0.79%	4	0.63%	12	1.89%	106	16.69%	501	78.90%	4	0.63%
Reason: No appointment necessary	3	0.47%	10	1.57%	4	0.63%	15	2.35%	85	13.32%	514	80.56%	7	1.10%

Statistical tests on differences in answers according to language region and level of education are provided in Additional Table 1. German-speaking respondents were more satisfied and stated they would recommend the service more, compared to French-speaking respondents. Statistically significant differences were also observed for the items ‘job increases risk for others’ (*p* < 0.001), ‘opening hours of pharmacies’(*p* = 0.03), ‘not having primary care physician’(*p* < 0.001), and ‘pricing’ (*p* = 0.01), which were less frequently mentioned as factors by French-speaking respondents, compared to the German-speaking respondents.

We observed that older than 65 years old customers had a stronger motivation to reduce others’ risk (*p* = 0.01), while younger than 65 years of age had a greater motivation because their job increases the risk for influenza (*p* < 0.001). No statistically significant differences in any of the items were observed when analysing by education levels. Statistical analyses by level of urbanity were not possible due to the small number of questionnaires from sub-urban communes (*n* = 20, 3%).

### Discussion

During the 2019/2020 influenza season, we identified that customer satisfaction with pharmacist-administered influenza vaccinations was high. Results from our voluntary questionnaire revealed that customers found the service to be comfortable and 99% were to recommend the service based on their experience. The primary reason for selecting the pharmacy for their influenza vaccination was convenience. The majority of respondents did get the seasonal influenza vaccination for the first time in a pharmacy (40.9%). Therefore, the results of this study suggest that expanding the role of pharmacists in Switzerland may be a means to improve overall access to vaccinations.

Pharmacist-led vaccination programs have emerged in the past decade as a means to improve access to the influenza vaccination within communities [5]. The vast majority of studies suggest patients are highly satisfied and comfortable with pharmacist-administered influenza vaccination programs. Our findings are concordant with previous studies at a national and international level. Within Switzerland, a 2018 survey conducted in the Swiss canton of Vaud analysed 301 questionnaires and identified that 94% of customers reported global satisfaction, 97% reported feeling comfortable with injection technique and provided information, and 84% would have recommended the service [14]. A similar survey performed in community pharmacies in Toronto, Canada, also reported high satisfaction with pharmacist-administered influenza vaccinations [11]. Of the 1502 surveyed customers in the Canadian study, 92% reported very high satisfaction with the pharmacists’ injection technique, 86% reported being very comfortable about being vaccinated by a pharmacist, and 99% would have recommended the service [11]. Similar results have been observed in Australia, where 99% of 434 customers were satisfied with the service overall and 97% stated to get community pharmacist-administered vaccinations in the future [12].

The survey in the canton of Vaud additionally collected positive qualitative comments about the easy access of vaccinations in pharmacies, which is in-line with our findings [14]. The majority of respondents (75.7%) in our study stated that the unavailability of a primary care physician was not the reason for choosing the pharmacies as a place for vaccination, suggesting customers actively chose pharmacies as an alternative option rather than being forced to do so. Rather, the main motivation in our survey was related to scheduling convenience. This was reflected by 96.2% of respondents indicating that ‘no need for an appointment’ and 92.6% stating ‘the opening hours of the pharmacies’ as motivational factors. Results from a customer survey in Nova Scotia, Canada, similarly identified convenience as a main reason for receiving the influenza vaccination in a pharmacy [13]. While we did not identify indicators of health inequality in our survey, a UK study identified that pharmacist-administered vaccination services were more likely to be accessed by individuals from more deprived areas where the convenience of longer opening hours may play an important role [23].

Contrary to receiving the vaccination by a primary care physician, vaccinations in the pharmacy without a physician’s prescription are not yet remunerated by the compulsory basic health insurance in Switzerland and customers need to have a voluntary supplementary insurance plan or pay for the service out-of-pocket. While this may hinder the uptake of the service, we identified that the majority of respondents (95%) answered positively when asked about the price. This indicates that the participating pharmacies applied prices which appear to be reasonable and justified to the customers, or the added value of convenience outweighs the cost.

One of the primary aims of pharmacist-administered vaccination services is to improve access to, and rates of, vaccination—particularly to high-risk patient groups. Steyer and colleagues were able to show that allowing American pharmacists to vaccinate customers improved influenza vaccine rates in individuals aged 65 years or older [24]. The greater likelihood of being immunised remained even when controlling for gender, ethnicity, income, health insurance, and health status. Pharmacists are skilled healthcare professionals that can be utilised to support healthcare systems, particularly in preventative medicine, such as vaccinations. It is pivotal that we consider the potential for the expanded role of pharmacists in communities to facilitate rapid and wide-spread access to vaccinations. While our study was not designed to compare vaccination rates, 13.5% of reported not having a primary care physician as a reason for choosing a pharmacy, suggesting an unmet need within the healthcare system. Moreover, while 85.7% of respondents stated that they would have gotten the vaccination elsewhere if not possible in the pharmacy, the high satisfaction rate suggests pharmacists are accepted healthcare providers within the community. Additionally, 32.5% of respondents stated that they had never received an influenza vaccination from a physician before, which may indicate that pharmacist-led services are an attractive means to reach new patients.

#### Limitations

There are several limitations of our study to be discussed. Most importantly, inclusion in the study was voluntary for both pharmacies and customers, which can lead to a selection bias. However, no incentive was provided to either the pharmacy or patient for their participation. Nevertheless, it is possible that participating pharmacies were very comfortable in providing vaccination services and were, hence, skilled providers. This could explain the high rate of satisfaction regarding the competence of the pharmacist reported in our study. Additionally, respondents in our survey seem to have been a highly motivated population favouring vaccinations altogether, as over 85% would have gone for the vaccination elsewhere if it were not for the pharmacy. A recent study identified that only 15% of Swiss individuals reported an influenza vaccination in the past 12 months [25]. Thus, our results are likely not generalisable to the entire Swiss population. Limitations on generalisability further include the geographical distribution of the participating pharmacies: certain cantons are not represented in the survey and participating pharmacies were mainly from urban communes. Additionally, we did not conduct a random sample of customers. Rather, the respondents were customers that had already actively chosen to be vaccinated in a pharmacy and, therefore, likely had a positive attitude regarding the service in general. Furthermore, while we protected the anonymity by providing sealable envelopes for respondents to place their completed surveys, it is possible responses may have been skewed to please their pharmacists. However, we believe this was likely minimal as a previous Swiss study revealed no difference between responses of regular customers and occasional customers on overall satisfaction and specific items (i.e., information, injection technique) [14]. Unsupervised questionnaire filling of Likert scales may introduce unintentional answers, as seen in the difference between having had done a previous influenza vaccination in a pharmacy and stating a positive previous experience as a reason for choosing the pharmacy as vaccination place. It is possible that the question relating to the previous positive experience was misinterpreted as having a positive experience with the pharmacy in general, rather than with a vaccination in the pharmacy, as intended. The low number of missing data and ‘not answerable’ options does, however, indicate appropriate questionnaire wording. Finally, we did not monitor the total number of vaccinations performed in the pharmacies, which hinders decisive conclusions on rates and selection.

### Conclusion

With this analysis of 656 customer surveys, we report high levels of satisfaction with community pharmacist-administered seasonal influenza vaccinations in Switzerland. This satisfaction included injection technique, used facilities, preparatory discussions, and pricing of the service. Easy scheduling appears to have been a primary driver for choosing pharmacies as the provider, not unavailability of primary care physicians. With the main age bracket of respondents being 65 to 74 years, community pharmacies seem to be able to help in fulfilling the seasonal influenza vaccination recommendations of the Federal Office of Public Health of Switzerland. Thus, the availability of pharmacist-administered services may be an important factor to improving the seasonal influenza vaccination rates in Switzerland.

## Supplementary information

**Additional file 1: Figure S1.** The German version of the questionnaire.

**Additional file 2: Table S1.** Statistical tests on differences in answers according to language region and level of education.

## Data Availability

The datasets used and/or analysed during the current study are available from the corresponding author on reasonable request.

## References

[1] Iuliano AD, Roguski KM, Chang HH, Muscatello DJ, Palekar R, Tempia S (2018). Estimates of global seasonal influenza-associated respiratory mortality: a modelling study. The Lancet..

[2] World Health Organization. Influenza (Seasonal) Fact Sheet [Internet]. WHO; 2018 [cited 2020 Apr 29]. Available from: https://www.who.int/news-room/fact-sheets/detail/influenza-(seasonal).

[3] Federal Office of Public Health of Switzerland. Empfehlung Grippeimpfung [Flu vaccination recommendation] [Internet]. Federal Office of Public Health; 2017 [cited 2020 Feb 3]. Available from: https://www.bag.admin.ch/bag/en/home/krankheiten/krankheiten-im-ueberblick/grippe.html.

[4] Federal Office of Public Health of Switzerland. BAG-Bulletin 29: Bericht zur Grippesaison 2018/19 [Report on the influenza season 2018/19] [Internet]. Federal Office of Public Health; 2019 [cited 2020 Feb 3]. Available from: https://www.bag.admin.ch/bag/en/home/krankheiten/krankheiten-im-ueberblick/grippe.html.

[5] Kirkdale CL, Nebout G, Megerlin F, Thornley T (2017). Benefits of pharmacist-led flu vaccination services in community pharmacy. Ann Pharm Fr..

[6] The Swiss Confederation. SR 811.11 Bundesgesetz über die universitären Medizinalberufe (Medizinalberufegesetz, MedBG) [Federal law on the university medical professions] [Internet]. 2015 [cited 2020 May 6]. Available from: https://www.admin.ch/opc/de/classified-compilation/20040265/index.html.

[7] The Swiss Confederation. Medizinalberuferegister [Register of Medical Professions] [Internet]. 2020 [cited 2020 May 6]. Available from: https://www.medregom.admin.ch/.

[8] pharmaSuisse. Kundenangebote Impfen und Impfberatung [Customer offers vaccination and vaccination advice] [Internet]. 2020 [cited 2020 May 6]. Available from: https://www.pharmasuisse.org/de/1159/Impfen-und-Impfberatung.htm.

[9] pharmaSuisse. Impfapotheken [Vaccination pharmacies] [Internet]. 2020 [cited 2020 May 6]. Available from: https://impfapotheke.ch/.

[10] Brunner I, Schmedders K, Wolfensberger A, Schreiber PW, Kuster SP. The economic and public health impact of influenza vaccinations: contributions of Swiss pharmacies in the 2016/17 and 2017/18 influenza seasons and implications for vaccination policy. Swiss Med Wkly [Internet]. 2019Dec 17 [cited 2020 Apr 24];149(5152). Available from: https://smw.ch/article/doi/smw.2019.20161. 10.57187/smw.2019.20161 32227800

[11] Papastergiou J, Folkins C, Li W, Zervas J (2014). Community pharmacist–administered influenza immunization improves patient access to vaccination. Can Pharm J Rev Pharm Can..

[12] Burt S, Hattingh L, Czarniak P (2018). Evaluation of patient satisfaction and experience towards pharmacist-administered vaccination services in Western Australia. Int J Clin Pharm..

[13] Isenor JE, Wagg AC, Bowles SK (2018). Patient experiences with influenza immunizations administered by pharmacists. Hum Vaccines Immunother..

[14] Vo N-LC, Du Pasquier S, Bugnon O. Enquête de satisfaction auprès des personnes vaccinées contre la grippe dans les pharmacies vaudoises durant l’automne 2018 [Master’s thesis]. [Lausanne]: University of Geneva; 2019.

[15] Krosnick JA, Fabrigar LR. Designing rating scales for effective measurement in surveys. In: Survey measurement and process quality [Internet]. John Wiley & Sons, Ltd; 2012 [cited 2019 May 28]. p. 141–64. Available from: https://onlinelibrary.wiley.com/doi/abs/10.1002/9781118490013.ch6.

[16] Electric Paper Informationssysteme GmbH. TeleForn (Data capturing software). Lüneburg: Electric Paper; 2019.

[17] Federal Statistical Office. Raumgliederungen [Spatial Divisions] [Internet]. 2020 [cited 2020 Feb 18]. Available from: https://www.bfs.admin.ch/.

[18] R Core Team. R: A language and environment for statistical computing [Internet]. Vienna, Austria: R Foundation for Statistical Computing; 2019 [cited 2019 Aug 14]. Available from: https://www.r-project.org/.

[19] Wickham H, Chang W, Henry L, Pedersen TL, Takahashi K, Wilke C, et al. ggplot2: create elegant data visualisations using the grammar of graphics [Internet]. 2020 [cited 2020 Apr 28]. Available from: https://CRAN.R-project.org/package = ggplot2.

[20] Wickham H, François R, Henry L, Müller K, RStudio. dplyr: A grammar of data manipulation [Internet]. 2019 [cited 2019 Jun 5]. Available from: https://CRAN.R-project.org/package = dplyr.

[21] Dowle M, Srinivasan A, Gorecki J, Chirico M, Stetsenko P, Short T, et al. data.table: Extension of ‘data.frame’ [Internet]. 2019 [cited 2020 Apr 28]. Available from: https://CRAN.R-project.org/package=data.table.

[22] Zumbach D. RSwissMaps: plot and save customised Swiss maps [Internet]. 2019 [cited 2020 Apr 28]. Available from: https://CRAN.R-project.org/package = RSwissMaps.

[23] Anderson C, Thornley T (2016). Who uses pharmacy for flu vaccinations? Population profiling through a UK pharmacy chain. Int J Clin Pharm..

[24] Steyer TE, Ragucci KR, Pearson WS, Mainous AG (2004). The role of pharmacists in the delivery of influenza vaccinations. Vaccine..

[25] Zürcher K, Zwahlen M, Berlin C, Egger M, Fenner L. Trends in influenza vaccination uptake in Switzerland: Swiss Health Survey 2007 and 2012. Swiss Med Wkly [Internet]. 2019 23 [cited 2020 Apr 27];149(0304). Available from: https://smw.ch/article/doi/smw.2019.14705. 10.4414/smw.2019.14705 30673116

