# Supplementary material for: Community pharmacist-administered seasonal influenza vaccination: a national customer survey
Source: J Pharm Policy Pract. 2020 Sep 25;13:57. doi: 10.1186/s40545-020-00259-7 (PMC7517795; doi:10.1186/s40545-020-00259-7)
Supplement: Supplementary file 1 — Additional file 1: Figure S1. The German version of the questionnaire. [file 40545_2020_259_MOESM1_ESM.zip › Additional Figure1_1_Influenza Survey_BMC.pdf]

2272149355

Kundenzufriedenheit Grippeimpfung Apotheke

Danke, dass Sie sich für eine Grippeimpfung bei uns in der Apotheke entschieden haben.

Unser Berufsverband, pharmaSuisse, möchte in der aktuellen Grippe-Saison zusammen mit der ETH Zürich mehr über die Motivation für eine Impfung in der Apotheke erfahren. Auch ist es uns und dem Verband ein Anliegen, Ihre Zufriedenheit mit dieser noch jungen Dienstleistung zu erfahren.

Der komplette Fragebogen kann innerhalb von ca. 5 Minuten ausgefüllt werden. Bitte füllen Sie den Fragebogen nach Ihrer Impfung in der Apotheke aus und legen Sie ihn in den beiliegenden Umschlag. Falls Sie eine Frage nicht beantworten können, weil die Frage beispielsweise nicht zu Ihrer konkreten Situation passt, können Sie “nicht beantwortbar” ankreuzen.

Ihre Antworten werden anonymisiert und durch die ETH Zürich automatisiert ausgewertet. Wir als Apotheke haben keine Einsicht in Ihre Originalantworten. Falls Sie uns als Apotheke eine Rückmeldung zur Impfung geben möchten, bitten wir Sie, uns diese direkt mitzuteilen.

Vielen Dank für Ihr Feedback!

Zu Beginn möchten wir allgemeine Informationen erfahren. Diese helfen uns bei der Auswertung.

In welcher Postleitzahl befindet sich die Apotheke, in welcher Sie die Grippeimpfung haben machen lassen? ☐☐☐☐

Wie lautet Ihre Anrede?

☐ Frau ☐ Herr

In welcher Alterskategorie befinden Sie sich?

<16 ☐ 16-17 ☐ 18-24 ☐ 25-34 ☐ 35-44 ☐ 45-54 ☐ 55-64 ☐ 65-74 ☐ 75-84 ☐ >85 ☐

Was ist Ihr höchster erworbener Ausbildungsgrad?

☐ Obligatorische Schule

☐ Sekundarstufe II: Berufsbildung (z. B. Lehre)

☐ Sekundarstufe II: Allgemeinbildung (z. B. Gymnasium)

☐ Höhere Berufsbildung (z. B. Höhere Fachschule)

☐ Hochschule (z. B. Universität, Fachhochschule)

Im Folgenden möchten wir Ihre Zufriedenheit mit der Dienstleistung erfahren. Bitte bewerten Sie, wie sehr folgende Aussagen auf Sie zutreffen.

Ich fühle mich wohl damit, dass eine Apothekerin / ein Apotheker die Impfung durchführt

☐ trifft überhaupt nicht zu ☐ trifft nicht zu ☐ trifft eher nicht zu ☐ trifft eher zu ☐ trifft zu ☐ trifft vollumfänglich zu ☐ nicht beantwortbar

Ich bin zufrieden mit der Injektionstechnik der Apothekerin / des Apothekers

☐ trifft überhaupt nicht zu ☐ trifft nicht zu ☐ trifft eher nicht zu ☐ trifft eher zu ☐ trifft zu ☐ trifft vollumfänglich zu ☐ nicht beantwortbar

Ich bin zufrieden mit der Besprechung meiner Bedenken bezüglich der Grippeimpfung

☐ trifft überhaupt nicht zu ☐ trifft nicht zu ☐ trifft eher nicht zu ☐ trifft eher zu ☐ trifft zu ☐ trifft vollumfänglich zu ☐ nicht beantwortbar

Ich bin zufrieden mit den Räumlichkeiten, welche für die Grippeimpfung verwendet werden

☐ trifft überhaupt nicht zu ☐ trifft nicht zu ☐ trifft eher nicht zu ☐ trifft eher zu ☐ trifft zu ☐ trifft vollumfänglich zu ☐ nicht beantwortbar

Ich bin zufrieden mit der Preisgestaltung der gesamten Dienstleitung

☐ trifft überhaupt nicht zu ☐ trifft nicht zu ☐ trifft eher nicht zu ☐ trifft eher zu ☐ trifft zu ☐ trifft vollumfänglich zu ☐ nicht beantwortbar

Ich würde die Grippeimpfung in der Apotheke weiterempfehlen

☐ trifft überhaupt nicht zu ☐ trifft nicht zu ☐ trifft eher nicht zu ☐ trifft eher zu ☐ trifft zu ☐ trifft vollumfänglich zu ☐ nicht beantwortbar
