# Supplementary material for: Community pharmacist-administered seasonal influenza vaccination: a national customer survey
Source: J Pharm Policy Pract. 2020 Sep 25;13:57. doi: 10.1186/s40545-020-00259-7 (PMC7517795; doi:10.1186/s40545-020-00259-7)
Supplement: Supplementary file 1 — Additional file 1: Figure S1. The German version of the questionnaire. [file 40545_2020_259_MOESM1_ESM.zip › Additional Figure1_2_Influenza Survey_BMC.pdf]

0045149351

Im Folgenden möchten wir Ihre Gründe für die Grippeimpfung erfahren.
Bitte bewerten Sie, wie sehr folgende Aussagen auf Sie zutreffen.

Ich möchte mein eigenes Risiko reduzieren, mich mit der Grippe anzustecken

☐ trifft überhaupt nicht zu ☐ trifft nicht zu ☐ trifft eher nicht zu ☐ trifft eher zu ☐ trifft zu ☐ trifft vollumfänglich zu ☐ nicht beantwortbar

Ich möchte andere Personen davor schützen, durch mich mit der Grippe angesteckt zu werden

☐ trifft überhaupt nicht zu ☐ trifft nicht zu ☐ trifft eher nicht zu ☐ trifft eher zu ☐ trifft zu ☐ trifft vollumfänglich zu ☐ nicht beantwortbar

Mein Beruf setzt mich einem höheren Risiko aus, mich mit der Grippe anzustecken

☐ trifft überhaupt nicht zu ☐ trifft nicht zu ☐ trifft eher nicht zu ☐ trifft eher zu ☐ trifft zu ☐ trifft vollumfänglich zu ☐ nicht beantwortbar

Ich arbeite mit Personen, welche vor einer Grippeinfektion geschützt werden müssen (z. B. Betagte, Kranke, Kinder)

☐ trifft überhaupt nicht zu ☐ trifft nicht zu ☐ trifft eher nicht zu ☐ trifft eher zu ☐ trifft zu ☐ trifft vollumfänglich zu ☐ nicht beantwortbar

Im Folgenden möchten wir Ihre Gründe für die Wahl einer Apotheke als Impfort erfahren.
Bitte bewerten Sie, wie sehr folgende Aussagen auf Sie zutreffen.

Weil mir die Öffnungszeiten der Apotheke entsprechen

☐ trifft überhaupt nicht zu ☐ trifft nicht zu ☐ trifft eher nicht zu ☐ trifft eher zu ☐ trifft zu ☐ trifft vollumfänglich zu ☐ nicht beantwortbar

Weil ich bezüglich Impfungen in der Apotheke bereits gute Erfahrungen gemacht habe

☐ trifft überhaupt nicht zu ☐ trifft nicht zu ☐ trifft eher nicht zu ☐ trifft eher zu ☐ trifft zu ☐ trifft vollumfänglich zu ☐ nicht beantwortbar

Weil ich keine Hausärztin/keinen Hausarzt habe

☐ trifft überhaupt nicht zu ☐ trifft nicht zu ☐ trifft eher nicht zu ☐ trifft eher zu ☐ trifft zu ☐ trifft vollumfänglich zu ☐ nicht beantwortbar

Weil meine Ärztin/mein Arzt nicht verfügbar ist

☐ trifft überhaupt nicht zu ☐ trifft nicht zu ☐ trifft eher nicht zu ☐ trifft eher zu ☐ trifft zu ☐ trifft vollumfänglich zu ☐ nicht beantwortbar

Weil der Preis für mich stimmt

☐ trifft überhaupt nicht zu ☐ trifft nicht zu ☐ trifft eher nicht zu ☐ trifft eher zu ☐ trifft zu ☐ trifft vollumfänglich zu ☐ nicht beantwortbar

Weil ich der Apotheke vertraue

☐ trifft überhaupt nicht zu ☐ trifft nicht zu ☐ trifft eher nicht zu ☐ trifft eher zu ☐ trifft zu ☐ trifft vollumfänglich zu ☐ nicht beantwortbar

Weil die Apotheke für mich leicht zugänglich ist

☐ trifft überhaupt nicht zu ☐ trifft nicht zu ☐ trifft eher nicht zu ☐ trifft eher zu ☐ trifft zu ☐ trifft vollumfänglich zu ☐ nicht beantwortbar

Weil ich ohne Termin (oder kurzfristig mit Termin) vorbeikommen kann

☐ trifft überhaupt nicht zu ☐ trifft nicht zu ☐ trifft eher nicht zu ☐ trifft eher zu ☐ trifft zu ☐ trifft vollumfänglich zu ☐ nicht beantwortbar

Würden Sie die Grippeimpfung auch anderswo durchführen lassen (falls in der Apotheke nicht möglich), z.B. bei einer Ärztin / einem Arzt?

☐ ja ☐ nein ☐ ich weiss es nicht

Wie oft haben Sie die Grippeimpfung bereits zuvor in einer Apotheke machen lassen?

☐ dieses war das erste Mal ☐ 1-2 Mal ☐ 3-4 Mal ☐ mehr als 4 Mal

Wie oft haben Sie die Grippeimpfung bereits zuvor bei einer Ärztin / einem Arzt machen lassen?

☐ nie ☐ 1-2 Mal ☐ 3-4 Mal ☐ mehr als 4 Mal

Haben Sie bereits eine andere Impfung (nicht Grippe) in der Apotheke machen lassen?

☐ ja ☐ nein

Bei Fragen zur Studie dürfen Sie sich melden bei:

ETH Zürich
Dr. phil. Dominik Stämpfli
Vladimir-Prelog-Weg 4
8093 Zürich

dominik.staempfli@pharma.ethz.ch
