# Supplementary material for: Community pharmacist-administered seasonal influenza vaccination: a national customer survey
Source: J Pharm Policy Pract. 2020 Sep 25;13:57. doi: 10.1186/s40545-020-00259-7 (PMC7517795; doi:10.1186/s40545-020-00259-7)
Supplement: Supplementary file 2 — Additional file 2: Table S1. Statistical tests on differences in answers according to language region and level of education. [file 40545_2020_259_MOESM2_ESM.docx]

Supplementary Table 1: Statistical tests on differences in answers according to language region and level of education.

|  | Language Differences* | | Education Differences | Differences between >=65 yo vs < 65 yo | |
| --- | --- | --- | --- | --- | --- |
| Variable | P-value | People agree more in | P-value | P-value | People agree more in |
| Satisfaction: Feeling comfortable | 0.30 |  | 0.38 | NaN |  |
| Satisfaction: Injection technique | NaN |  | NaN | NaN |  |
| Satisfaction: Talk about my concerns | 0.61 |  | 0.36 | 0.44 |  |
| Satisfaction: Facilities | 0.31 |  | 0.38 | 0.60 |  |
| Satisfaction: Pricing | 0.47 |  | 0.70 | 0.85 |  |
| Satisfaction: I would recommend | 0.05 | GER | 0.82 | 0.19 |  |
| Motivation: Reduce my own risk | 0.78 |  | 0.25 | 0.30 |  |
| Motivation: Reduce others' risk | 0.12 |  | 0.70 | 0.01 | >= 65 yo |
| Motivation: Job increases risk | 0.07 |  | 0.22 | 0.00 | < 65 yo |
| Motivation: Job increases risk for others | 0.00 | FR | 0.19 | 0.45 |  |
| Reason: Opening hours of pharmacy | 0.03 | FR | 0.66 | 0.29 |  |
| Reason: Positive experience in the past | 0.09 | FR | 0.29 | 0.29 |  |
| Reason: No primary care physician | 0.00 | FR | 0.22 | 0.56 |  |
| Reason: Availability of physician | 0.48 |  | 0.72 | 0.19 |  |
| Reason: Pricing | 0.01 | FR | 0.40 | 0.69 |  |
| Reason: Trust in the pharmacy | 0.38 |  | 0.67 | 0.19 |  |
| Reason: Accessibility of the pharmacy | 0.08 |  | 0.07 | 0.55 |  |
| Reason: No appointment necessary | 0.38 |  | 0.68 | 0.52 |  |
| * Refers to questionnaire language. | | | | | |
| Mann-Whitney U Test to compare the null hypothesis of no differences between French and German questionnaires regions; Kruskal-Wallis test to compare the null hypothesis of no differences between different levels of education. A significant p-value (p <0.05) indicates that there are statistically significant differences on the responses. | | | | | |
